# Use of Attribute Driven Incremental Discretization and Logic Learning Machine to build a prognostic classifier for neuroblastoma patients

**DOI:** 10.1186/1471-2105-15-S5-S4

**Published:** 2014-05-06

**Authors:** Davide Cangelosi, Marco Muselli, Stefano Parodi, Fabiola Blengio, Pamela Becherini, Rogier Versteeg, Massimo Conte, Luigi Varesio

**Affiliations:** 1 Laboratory of Molecular Biology, Gaslini Institute, Largo Gaslini 5, 16147 Genoa, Italy; 2 Institute of Electronics, Computer and Telecommunication Engineering, National Research Council of Italy, Genoa 16149, Italy; 3 Department of Human Genetics, Academic Medical Center, University of Amsterdam, Meibergdreef 15, Amsterdam 1100, The Netherlands; 4 Department of Hematology-Oncology, Gaslini Institute, Largo Gaslini 5, Genoa 16147, Italy

**Keywords:** Logic Learning Machine, Attribute Driven Incremental Discretization, Explicit rules, NB-hypo-II signature, Neuroblastoma, Hypoxia, Classifier, Weighted Classification

## Abstract

**Background:**

Cancer patient's outcome is written, in part, in the gene expression profile of the tumor. We previously identified a 62-probe sets signature (NB-hypo) to identify tissue hypoxia in neuroblastoma tumors and showed that NB-hypo stratified neuroblastoma patients in good and poor outcome [1]. It was important to develop a prognostic classifier to cluster patients into risk groups benefiting of defined therapeutic approaches. Novel classification and data discretization approaches can be instrumental for the generation of accurate predictors and robust tools for clinical decision support. We explored the application to gene expression data of Rulex, a novel software suite including the Attribute Driven Incremental Discretization technique for transforming continuous variables into simplified discrete ones and the Logic Learning Machine model for intelligible rule generation.

**Results:**

We applied Rulex components to the problem of predicting the outcome of neuroblastoma patients on the bases of 62 probe sets NB-hypo gene expression signature. The resulting classifier consisted in 9 rules utilizing mainly two conditions of the relative expression of 11 probe sets. These rules were very effective predictors, as shown in an independent validation set, demonstrating the validity of the LLM algorithm applied to microarray data and patients' classification. The LLM performed as efficiently as Prediction Analysis of Microarray and Support Vector Machine, and outperformed other learning algorithms such as C4.5. Rulex carried out a feature selection by selecting a new signature (NB-hypo-II) of 11 probe sets that turned out to be the most relevant in predicting outcome among the 62 of the NB-hypo signature. Rules are easily interpretable as they involve only few conditions.

Furthermore, we demonstrate that the application of a weighted classification associated with the rules improves the classification of poorly represented classes.

**Conclusions:**

Our findings provided evidence that the application of Rulex to the expression values of NB-hypo signature created a set of accurate, high quality, consistent and interpretable rules for the prediction of neuroblastoma patients' outcome. We identified the Rulex weighted classification as a flexible tool that can support clinical decisions. For these reasons, we consider Rulex to be a useful tool for cancer classification from microarray gene expression data.

## Background

Neuroblastoma (NB) is the most common solid pediatric tumor, deriving from ganglionic lineage precursors of the sympathetic nervous system [2]. It shows notable heterogeneity of clinical behavior, ranging from rapid progression, associated with metastatic spread and poor clinical outcome, to spontaneous, or therapy-induced regression into benign ganglioneuroma. Age at diagnosis, stage and amplification of the N-myc proto-oncogene (*MYCN*) are clinical and molecular risk factors that the International Neuroblastoma Risk Group (INRG) utilized to classify patients into high, intermediate and low risk subgroups on which current therapeutic strategy is based. About fifty percent of high-risk patients die despite treatment making the exploration of new and more effective strategies for improving stratification mandatory [3].

The availability of genomic profiles improved our prognostic ability in many types of cancers [4]. Several groups used gene expression-based approaches to stratify NB patients. Prognostic gene signatures were described [5-11] and classifier proposed to predict the risk class and/or patients' outcome [5-13]. We and other scientific groups have identified tumor hypoxia as a critical component of neuroblastoma progression [14-16]. Hypoxia is a condition of low oxygen tension occurring in poorly vascularized areas of the tumor which has profound effects on cell growth, genotype selection, susceptibility to apoptosis, resistance to radio- and chemotherapy, tumor angiogenesis, epithelial to mesenchymal transition and propagation of cancer stem cells [17-20]. Hypoxia activates specific genes encoding angiogenic, metabolic and metastatic factors [18,21] and contributes to the acquisition of the tumor aggressive phenotype [18,22,23]. We have used gene expression profile to assess the hypoxic status of NB cells and we have derived a robust 62-probe sets NB hypoxia signature (NB-hypo) [14,24], which was found to be an independent risk factor for neuroblastoma patients [1].

The use of gene expression data for tumor classification is hindered by the intrinsic variability of the microarray data deriving from technical and biological variability. These limitation can be overcome by analyzing the results through algorithms capable to discretize the gene expression data in broad ranges of values rather than considering the absolute values of probe set expression. We will focus on the discretization approach to deal with gene expression data for patients' stratification in the present work.

Classification is central to the stratification of cancer patients into risk groups and several statistical and machine learning techniques have been proposed to deal with this issue [25]. We are interested in classification methods capable of constructing models described by a set of explicit rules for their immediate translation in the clinical setting and for their easily interpretability, consistency and robustness verification [15,26]. A rule is a statement in the form "if<premise> then<consequence>" where the premise is a logic product (AND) of conditions on the attributes of the problem and the consequence indicates the predicted output. Most used rule generation techniques belong to two broad paradigms: decision trees and methods based on Boolean function synthesis.

The decision tree approach implements discriminant policies where differences between output classes are the driver for the construction of the model. These algorithms divide iteratively the dataset into smaller subsets according to a divide and conquer strategy, giving rise to a tree structure from which an explicit set of rules can be easily retrieved. At each iteration a part of the training set is split into two or more subsets to obtain non-overlapping portions belonging to the same output class [27]. Decision tree methods provide simple rules, and require a reduced amount of computational resources. However, the accuracy of the models is often poor. The divide and conquer approach prevents the applicability of these models to relatively small datasets that would be progressively fractionated in very small, poorly indicative, subsets.

Methods based on Boolean function synthesis adopt an aggregative policy where some patterns belonging to the same output class are clustered to produce an explicit rule at any iteration. Suitable heuristic algorithms [28-30] are employed to generate rules exhibiting the highest covering and the lowest error; a tradeoff between these two objectives has been obtained by applying the Shadow Clustering (SC) technique [28] which leads to final models, called Logic Learning Machines (LLM), exhibiting good accuracy. The aggregative policy can also consider patterns already included in previously built rules; therefore, SC generally produces overlapping rules that characterize each output class better than the divide-and-conquer strategy. Clustering samples of the same kind permits to extract knowledge regarding similarities of the members of a given class rather than information on their differences. This is very useful in most applications and leads to models showing higher generalization ability, as shown by trials performed with SC [31,32].

LLM algorithms prevent the excessive fragmentation problem typical of divide-and-conquer approach but come at the expense of the need to implement an intelligent strategy for managing conflicts occurring when one instance is satisfied by more than one rules classifying opposite outcomes. LLM is a novel and efficient implementation of the Switching Neural Network (SNN) model [33] trained through an optimized version of the SC algorithm. LLM, SNN and SC have been successfully used in different applications: from reliability evaluation of complex systems [34] to prediction of social phenomena [35], form bulk electric assessment [36] to analysis of biomedical data [15,31,32,37,38].

The ability of generating models described by explicit rules has several advantages in extracting important knowledge from available data. Identification of prognostic factors in tumor diseases [15,37] as well as selection of relevant features in microarray experiments [31] are only two of the valuable targets achieved through the application of LLM and SNN. In this analysis, to improve the accuracy of the model generated by LLM, a recent innovative preprocessing method, called Attribute Driven Incremental Discretization (ADID) [39] has been employed. ADID is an efficient data discretization algorithm capable of transforming continuous attributes into discrete ones by inserting a collection of separation points (cutoffs) for each variable. The core of ADID consists in an incremental algorithm that adds the cutoff iteratively obtaining the highest value of a quality measure based on the capability of separating patterns of different classes. Smart updating procedures enable ADID to efficiently get a (sub) optimal discretization. Usually, ADID produces a minimal set of cutoffs for separating all the patterns of different classes. ADID and LLM algorithms are implemented in Rulex 2.0 [40], a software suite developed and commercialized by Impara srl that has been utilized for the present work.

Blending the generalization and the feature selection strength of LLM and the efficiency of ADID in mapping continuous variables into a discrete domain with the stratification power of the NB-hypo signature we obtained an accurate predictor of NB patients' outcome and a robust tool for supporting clinical decisions. In the present work, we applied Rulex 2.0 components to the problem of classifying and predicting the outcome of neuroblastoma patients on the bases of hypoxia- specific gene expression data. We demonstrate that our approach generates an excellent discretization of gene expression data resulting in a classifier predicting NB patients' outcome. Furthermore, we show the flexibility of this approach, endowed with the ability to steer the outcome towards clinically oriented specific questions.

## Results

### Rulex model

We analyzed gene expression of 182 neuroblastoma tumors profiled by the Affymetrix platform. The characteristics of the NB patients are shown in Table 1 and are comparable to what previously described [6]. We selected this dataset because the gene expression profile of the primary tumor, performed by microarray, was available for each patient. "Good" or "poor" outcome are defined, from here on, as the patient's status "alive" or "dead" 5 years after diagnosis respectively.

**Table 1:** Characteristics of 182 neuroblastoma patients included in the study.

Risk factors and outcome		Training set (n = 109)^a^%		Independent test set (n = 73)^a^	
		**Number**	%	**Number**	%

**Age at diagnosis (Years)**	1	54	49	33	46
	**>**1	55	51	40	54
**INSS stage**					
	1	29	26	14	19
	2	15	14	9	13
	3	16	15	7	9
	4	33	30	35	48
	4s	16	15	8	11
**MYCN status**					
	Normal	91	83	61	83
	Amplified	18	17	12	17
**Outcome**					
	Good	81	74	50	68
	Poor	28	26	23	32

^a ^The number of patients is 109 in the training set and 73 in the test set. The data show the total number of patients and the relative percentage in each subdivision.

**Figure 1:**
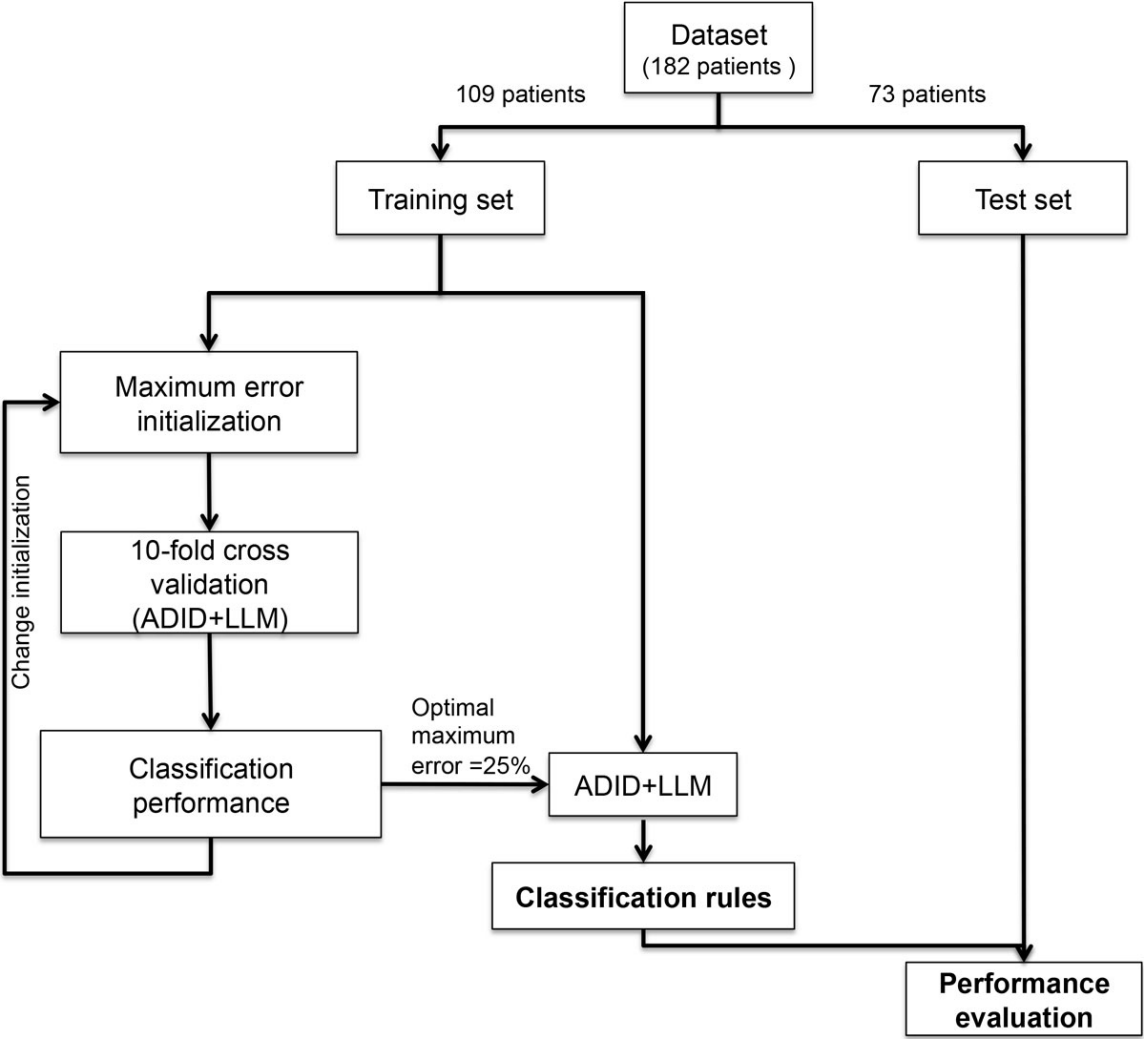
**Rule generation workflow**. The initial 182 patients dataset is randomly divided into training and test sets. The training set is used by the supervised learning procedure to iteratively calculate the LLM parameter:" maximum error allowed for a rule" by performing a complete 10-fold cross validation. The whole training set is randomly subdivided into 10 non-overlapping subsets, nine of which are used to train the classifier by employing ADID and LLM. The classifier is subsequently used to predict the outcome of the patients in the excluded subset. This procedure is repeated 10 times until every subset is classified once. Each parameter value is then evaluated according to the mean classification accuracy obtained in the cross validation. The parameter value, which obtained the highest mean accuracy, is selected to generate the final optimal classification rules. The rules are then tested on an independent cohort to assess their ability to predict patients' outcome.

We previously described the NB-hypo 62 probe sets signature that represents the hypoxic response of neuroblastoma cells [14,24] and used this signature for developing the hypoxia-based classifier to predict the patients' status utilizing ADID to convert the continuous probe sets values into discrete attributes and LLM algorithm to generate classification rules. Both techniques are implemented in Rulex 2.0. The first assessment of the classifier was done on the training set of 109 randomly chosen patients, while the remaining 73 patients were utilized to validate the predictions (Figure 1). The outcome of the classifier is a collection of rules, in the form if<premise> then<consequence>, where the premise includes conditions based on the probe sets values and the consequence is the patient status. Rulex 2.0 will use these rules collectively for outcome prediction on the validation set.

The generation of the classification rules requires a discretization step because this simplifies the selection of the cut-off values of the probe sets expression (Figure 1). The discretization yielded one cutoff value for each probe set which was sufficient for modeling the outcome. The use of a single cutoff dichotomizes the probe set attributes in low or high expression, drastically reducing the influence of the technical and biological variability present in the models associated with the absolute values of probe sets expression.

Furthermore, a test on the maximum error allowed for a rule was defined. The final classification rules were trained with the optimal value of 25% associated with the maximal mean accuracy of 87%, determined by 10 fold cross validation analysis (Figure 1). The procedure generated 9 rules, numbered from 1 to 9 (Rule ID) in Table 2 and based on conditions containing high or low probe sets expression value (above or below the set threshold respectively). Rule premises are limited to two conditions with the exception of rule 7 that has only one condition. Six rules predict good outcome and 3 poor outcome; they will be considered together in scoring the class attribution of new patients of the validation set. This is the optimal scenario proposed by Rulex 2.0 to utilize the 62 probe sets NB-hypo signature for classifying patients' outcome.

**Table 2:** Classification rules.

Rule ID^a^		Cond 1	Cond 2		Predicted Outcome	Covering^b^ (%)	Error^c^ (%)	Fisher pvalue^d^
**1**	*IF(*	217356_s_at ≤ 721	226452_at < 326	*)THEN*	Good	80	3.5	<0.001
**2**	*IF (*	206686_at ≤ 26	226452_at ≤326	*)THEN*	Good	70	14	<0.001
**3**	*IF (*	200738_s_at ≤ 1846	230630_at**>**23	*)THEN*	Good	62	10	<0.001
**4**	*IF(*	209446_s_at ≤ 57	223172_s_at **<**73	*)THEN*	Good	60	10	<0.001
**5**	*IF(*	202022_at > 131	223193_x_at < 324	*)THEN*	Good	60	14	<0.001
**6**	*IF(*	224314_s_at ≤ 29	236180_at <13	*)THEN*	Good	48	7.1	<0.001
**7**	*IF(*	217356_s_at > 721		*)THEN*	Poor	92	17	<0.001
**8**	*IF(*	223172_s_at >73	226452_at> 326	*)THEN*	Poor	60	8.6	<0.001
**9**	*IF(*	206686_at > 26	223172_s_at>73	*)THEN*	Poor	57	7.4	<0.001

^a ^Cond 1 and Cond 2 indicate the conditions into the premises of the rules.

^b ^The covering accounts for the fraction of patients that verify the rule and belong to the target outcome.

^c ^The error accounts for the fraction of patients that satisfy the rule and do not belong to the target outcome.

^d ^Fisher p-value quantifies the statistical significance of the rule on the basis of the number of patients correctly and incorrectly classified by a rule and the number of patients of the dataset belonging to each specific outcome.

**Figure 2:**
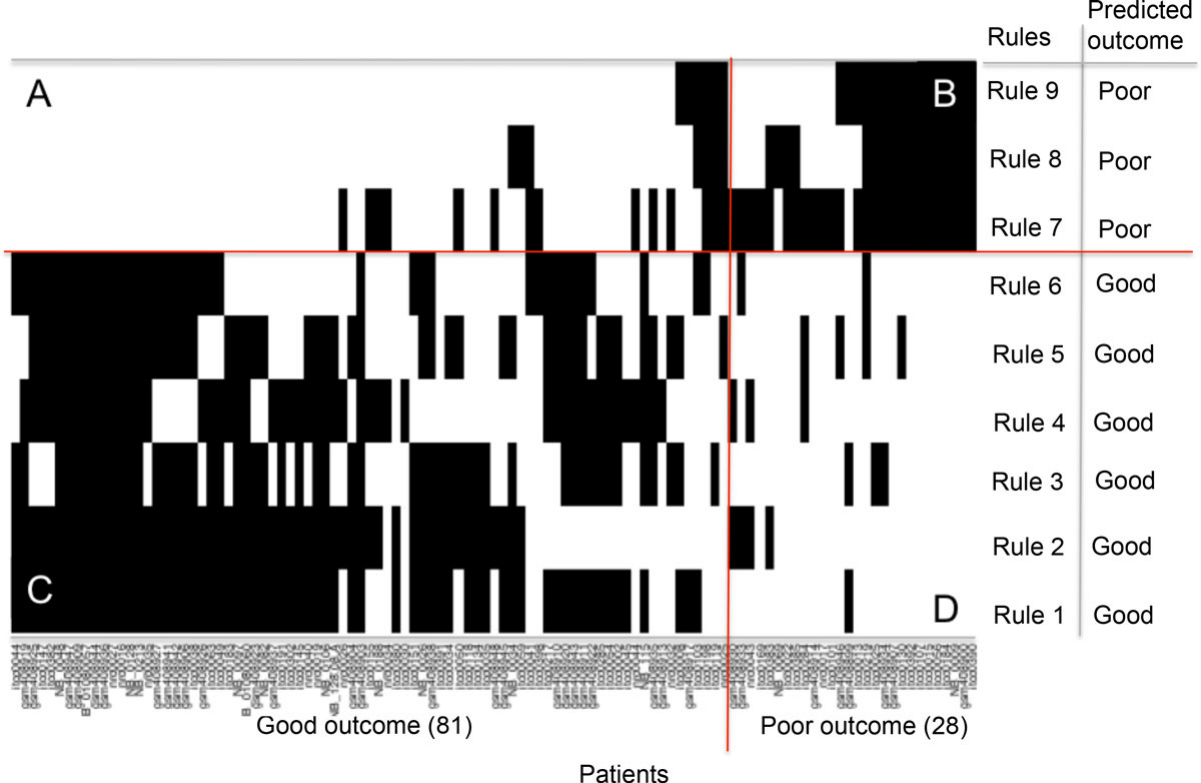
**Patients representation in the rules of Table 2**. Plot of the membership of the 109 patients (x axis) to the rules (y axis) of the classifier in Table 2. The rule identifier and predicted outcome are listed in right side of the plot. Two oriented lines divide the plot. The horizontal line separates the rules classifying good outcome from those classifying poor outcome. The vertical line separates good outcome from poor outcome patients. Each point of the plot indicates the membership of a patient to one rule. The two lines separate the plot in four sections labeled as A, B, C and D. Section A includes all the patients incorrectly classified by a poor outcome rule. Section B includes all the patients correctly classified by a poor outcome rule. Section C includes all the patients correctly classified by a good outcome rule and Section D includes all the patients incorrectly classified by a good outcome rule.

Three parameters, shown in Table 2, estimate the quality of the rules: 1) covering, measuring the generality, 2) error, measuring the ambiguity and 3) Fisher's *p*-value measuring the significance of each rule. The statistical significance of each rule by Fisher's exact test was very high (*p*< 0.001) providing strong evidence of the excellent quality of the rules. The covering ranged among rules from 48% (rule 6) to 80% (rule 7) for good outcome classes and ranged from 57% (rule 9) to 92% (rule 7) for poor outcome. Error ranged from 3.5% (rule 1) to 14% (rules 2 and 5) for good outcome class and ranged from 7.4% (rule 9) to 17% (rule 7) for poor outcome class. These rules have interesting features that will be addressed in detail. The first consideration is that the overall covering of the rules classifying good and poor outcome adds up to 380% and 209%, respectively, indicating overlap among rules. This is a characteristic of the LLM method implementing an aggregative, rather than fragmentation, policy as illustrated in the Materials and Methods section. However, overlap among the rules can lead to a conflict if the probe sets values of a patient satisfies two or more rules predicting opposite outcomes. To investigate whether overlapping among our rules can be source of conflicts we plotted in Figure 2 each patient's membership to the nine rules. The plot clusters the rules classifying good and poor outcome and the patients belonging to good or poor outcome classes. The results show that each patient is covered by at least one rule. Overlaps exist and occur mainly among rules predicting the same outcome (Figure 2B and 2C), which do not lead to classification conflicts. However, 30 patients (27% of the dataset) were covered by rules pertaining to opposite outcomes and are represented by those present in quadrant 2A (19 patients) or 2D (11 patients). Rulex 2.0 overcomes this problem by employing for assigning a class to a new sample a fast procedure that evaluates all the rules satisfied by it and their covering, thus generating a consensus outcome to be assigned to the sample as detailed in the Materials and Methods section.

A second characteristic of the classification rules is that they include only 11 out of 62 probe sets of the original NB-hypo signature. The relationship among probe sets and rules is shown in Table 3. Rulex 2.0 operated a second feature selection on the original 62 probe sets optimized for functioning in a binary profile of low and high probe set expression and gave rise to a modified hypoxia signature that we name NB-hypo-II.

**Table 3:** Probe sets characteristics of the new NB-hypo-II signature.

Probe sets^a^	Rule ID^b^	Probe set relevance^c^	
		**Good outcome**	**Poor outcome**

200738_s_at	3	-0.49	0
202022_at	5	0.5	0
206686_at	2, 9	-0.48	0.26
209446_s_at	4	-0.25	0
217356_s_at	1, 7	-0.74	0.92
223172_s_at	4, 8, 9	-0.35	0.48
223193_x_at	5	-0.1	0
224314_s_at	6	-0.22	0
226452_at	l, 2, 8	-0.26	0.34
230630_at	3	0.13	0
236180_at	6	-0.25	0

^a ^Affymetrix probe sets belonging to NB-hypo-II signature.

^b ^Rule ID indicates the ID of the rules in which the probe set occurs (as illustrated in Table 2).

^c ^Relevance measures the importance of the features included into the rules.

The relevance calculated for each outcome is shown.

It was of interest to assess the relative importance of each probe set in the classification scheme leading to identification of poor and good outcome patients. Negative or positive values indicate low or high expression associated with the predicted outcome and the relative relevance is measured by the absolute value. Ranking the probe sets may be of relevance to pick the genes for further validation on an alternative platform. It is interesting to note that probe set 217356_s_at is the most relevant for the classification of good and poor outcome patients.

A third interesting feature of the rules is the dichotomization of the expression values of each probe set even if Rulex 2.0 had no constraints on the discretization that could lead to multiple cutoffs or overlapping expression values in different rules. We were intrigued by these results and decided to examine the relationship between the cutoff expression values identified by Rulex and those obtained by Kaplan-Meyer analysis. The Kaplan-Meier algorithm calculates all possible cut- off points of a given probe set in a cohort of patients and selects the one maximizing the distinction between good and poor outcome. The results, shown in Table 4, demonstrated a general quite good concordance between the cutoff values of the Kaplan-Meier and those generated by Rulex. In particular, the two measures always differed by less than ±50% in magnitude, but only in some cases (probe sets 2, 3, 4, 5, and 8 for both overall and relapse free survival, and probe sets 6 and 7 for overall survival such a difference was lower than 25%. These (even if rather small) discrepancies are probably related to the capability of ADID to exploit the complex multivariate correlation among probe sets. Furthermore, we found a concordance also in the relationship between high/low probe set and outcome in Rulex derived rules and Kaplan-Meier plots (Table 4).

**Table 4:** Expression cut-offs from the Kaplan-Meier and from the rules.

Probe set ID^a^	NB-hypo-II^b^	Overall^c^				Relapse free^d^			
		
		Expression cut-off^e^		Worse^f^		Expression cut-off^e^		Worse^f^	
		**Kaplan-Meier**	**Rulex**	**Kaplan-Meier**	**Rulex**	**Kaplan-Meier**	**Rulex**	**Kaplan-Meier**	**Rulex**
		
1	223172_s_at	107	73	high	high	107	73	high	high
2	200738_s_at	1553	1846	high	high	1553	1846	high	high
3	209446_s_at	69	57	high	high	69	57	high	high
4	226452_at	280	326	high	high	280	326	high	high
5	217356_s_at	706	721	high	high	706	721	high	high
6	236180_at	18	13	hgih	hgih	13	13	high	hgih
7	202022_at	101	131	low	low	138	131	low	low
8	224314_s_at	25	29	high	high	25	29	high	high
9	206686_at	36	26	high	high	36	26	high	high
10	223193_x_at	495	324	high	high	572	324	high	high
11	230630_at	19	23	low	low	35	23	low	low

^a ^Probe sets ID indicates the numerical identified of the probeset.

^b ^NB-hyp-II indicates the list of probe sets of the NB-hypo signature belonging to the rules.

^c ^Overall indicates the survival time between the time of an event or last follow up and the time of diagnosis.

^d ^Relapse free indicates the survival time between the first relapse and the time of diagnosis.

^e ^Expression cut-off indicates the optimal cut-off point of each probe set resulting from the Kaplan-Meier scan and from the rules.

^f ^Worse indicates whether high or low expression of a given probe set is associated to the worse survival. Worse survival are calculated from the Kaplan-Meier curve or from the conditions included into the rules.

### Outcome prediction

The ability of the rules in Table 2 to predict patients' outcome was tested on a 73 patients' independent dataset. Results are expressed as accuracy, recall and precision, assessing the performance in classifying good outcome, specificity and negative predictive values (NPV) assessing the performance in classifying poor outcome patients. The direct evaluation of the performance of the 9 rules on the validation set is represented by ADID in Table 5, by ADID+LLM in Table 6, the base configuration of Table 7 and by no dataset modification in Additional File 1. The results demonstrate a good accuracy comparable to what reported by other algorithms [13]. Furthermore, the classification of good outcome was superior to that of poor outcome patients as shown for example by a recall of 90% relative to 57% of specificity.

**Table 5:** Comparison among discretization algorithms' performance.

Algorithm^f^	Accuracy^a^	Recall^b^	Precision^c^	Specificity^d^	NPV^e^
**ADID**	80%	90%	82%	57%	72%
**EntMDL**	68%	60%	91%	87%	50%
**Modified Chi2**	71%	64%	91%	87%	53%
**ROC-based**	77%	84%	82%	61%	64%
**Equal Frequency**	68%	60%	91%	87%	50%

^a ^Accuracy is the fraction of correctly classified patients and overall classified patients.

^b ^Recall is the fraction of correctly classified good outcome patients and the overall predicted good outcome patients.

^c ^Precision is the fraction of correctly classified good outcome patients and the predicted good outcome patients.

^d ^Specificity is the fraction of correctly classified poor outcome patients and the overall poor outcome patients.

^e ^NPV(negative predictive value) is the fraction of correctly classified poor outcome patients and the overall predicted poor outcome patients.

^f ^Discretization algorithms utilized for comparison/

**Table 6:** Performance of classification algorithms.

Algorithm^f^	Accuracy^a^	Recall^b^	Precision^c^	Specificity^d^	NPV^e^
**ADID + LLM**	80%	90%	82%	57%	72%
**Decision tree**	63%	76%	72%	35%	40%
**PAM**	81%	90%	83%	61%	74%
**SVM**	84%	94%	84%	61%	82%

^a ^Accuracy is the fraction of correctly classified patients and overall classified patients.

^b ^Recall is the fraction of correctly classified good outcome patients and the overall predicted good outcome patients.

^c ^Precision is the fraction of correctly classified good outcome patients and the predicted good outcome patients.

^d ^Specificity is the fraction of correctly classified poor outcome patients and the overall poor outcome patients.

^e ^NPV(negative predictive value) is the fraction of correctly classified poor outcome patients and the overall predicted poor outcome patients.

^f ^Machine learning algorithms utilized for comparison.

**Table 7:** Performance comparison among the configurations in the weighted classification on the test set.

Configuration ^f^	Accuracy^a^	Recall^b^	Precision^c^	Specificity^d^	Negative Predictive Value^e^
**Base (not weighted)**	80%	90%	82%	57%	72%
**W26_74 (balanced outcome)**	65%	63%	82%	70%	47%
**Wl_1000 (bias poor)**	66%	60%	86%	78%	47%
**W1000_1 (bias good)**	78%	98%	77%	35%	89%

^a ^Accuracy is the fraction of correctly classified patients and overall classified patients.

^b ^Recall is the fraction of correctly classified good outcome patients and the overall predicted good outcome patients.

^c ^Precision is the fraction of correctly classified good outcome patients and the predicted good outcome patients.

^d ^Specificity is the fraction of correctly classified poor outcome patients and the overall poor outcome patients.

^e ^Negative predictive value is the fraction of correctly classified poor outcome patients and the overall predicted poor outcome patients.

^f ^Configuration indicates the specific weights assigned to the outcomes in the weighted classification.

PVCA analysis [41] was utilized to estimate the potential variability of experimental effects including batch. The analysis revealed that batch effect explained a moderate 21% of the overall variation in our dataset and a Frozen Surrogate Variable Analysis (FSVA) was employed for removing batch effect. The application of FSVA reduced batch effect to less than 0.05% of the total variation (data not shown).

We compared the performances achieved by ADID and LLM on the batch-adjusted dataset and those on the original dataset (no dataset modification) to measure the impact of batch effect on classifier performances. Performance obtained with the adjusted dataset turns out to be very similar to that obtained with original data as shown in Additional file 1 demonstrating that batch effect had negligible impact on the performances. Therefore, the dataset with no modifications was utilized for subsequent analysis.

We then compared the performance of the ADID discretization approach and those of commonly used discretization algorithms, namely: entropy based (EntMDL [42]), Modified Chi Square [43], ROC based (Highest Youden index([44]), and Equal frequency (i.e. median expression for each feature). Results detailed in Table 5 showed that the discretization performed by ADID produced better accuracy with respect to the others (80% vs. 68%-77%).

We also compared the performance of LLM and those of Decision Tree [45], Support Vector Machines (SVM) [46], and Prediction Analysis of Microarrays (PAM) [47], to evaluate the ability of LLM to predict patients' outcome with respect to other standard supervised learning methods. Results in Table 6 revealed that ADID and LLM were able to predict patients' outcome with better performances with respect to the decision tree classifier. The performances of LLM, PAM and SVM classifiers were comparable.

Overall, the performance was good but unbalanced datasets tend to bias the performance towards the most represented class [48]. In our dataset the good outcome patients were more frequent (26 % poor and 74% good outcome). Therefore, we explored the possibility of utilizing a weighted classification system (WCS) [48-53] to improve the classification of poorly represented classes or to force the algorithm to maximize the performance on selected outcomes. The performance of the base configuration was taken as reference.

We performed a weighted classification accounting for the unbalanced class representation in the dataset (Table 7 configuration W26_74). The weight was calculated as the inverse proportion of the number of patients belonging to each class, about 3 times more weight on the poor outcome class. The major improvement over the base configuration was the specificity whereas all other parameters were similar or worst. Configuration W1_1000, was similar to W26_74, but set the weight of poor outcome 1000 times higher than that of the good one. Interestingly, its performance was very close to that of configuration W26_74 despite the disparity in the weight applied, suggesting that small changes in the relative weight of poor outcome are sufficient to optimize the results. In conclusion, increased weight on poor outcome augmented the percentage of correctly classified poor outcome patients even though a smaller number of patients were included in this class. This correction may be relevant when maximization of the specificity isof primary importance as in the case of using a prudent therapy.

In contrast, configuration W1000_1 sets the weight of good outcome 1000 times higher than that of poor outcome. The performance parameters were similar or higher than the base configuration with the exception of specificity that was quite low, a situation that appears symmetrical to those observed previously. The recall is almost absolute indicating the exceptional ability to classify good outcome. The drawback of this configuration is a very low percentage of correct poor outcome classification that is 35% of all poor outcome patients. This configuration may be useful in the case of using an aggressive therapy.

In conclusion, WCS can improve performance parameters of classification of poor or good outcome patients and may be particularly relevant in a situation where the dataset contains a major unbalance between classes and/or when clinical decisions may require minimizing false positives or false negatives.

## Discussion

Our study is based on gene expression data derived through the analysis of primary neuroblastoma tumors by microarray on the Affymetrix platform. We focused on the expression of 62 probe sets comprising the NB-hypo signature that we have previously shown to represent the hypoxia status of neuroblastoma cells [24]. The association of hypoxia with poor prognosis in neuroblastoma patients was previously demonstrated [16,54]. We studied a cohort of 182 NB patients characterized by clinical and molecular data addressing the question of the potential prognostic value of this signature. Rulex 2.0 suite was used to train a model on a set of patients and validate it on an independent one. We demonstrate that Attribute Driven Incremental Discretization and Logic Learning Machine algorithms, implemented in Rulex 2.0, generated a robust set of rules predicting outcome of neuroblastoma patients using expression values of 11 probe sets, specific for hypoxia extracted from the gene NB-hypo expression profile.

Outcome prediction of NB patients was reported by several groups using a combination of different risk factors and utilizing various algorithms [5,6,12,55,56]. Several groups have used gene expression-based approaches to stratify neuroblastoma patients and prognostic gene signatures have been described often based on the absolute values of the probe sets after appropriate normalization [5-11,13]. Affymetrix GeneChip microarrays are the most widely used high-throughput technology to measure gene expression, and a wide variety of preprocessing methods have been developed to transform probe intensities reported by a microarray scanner into gene expression estimates [57]. However, variations from one experiment to another [58] may increase data variability and complicate the interpretation of expression analysis based on absolute gene expression values. We addressed the problem by applying the Attribute Driven Incremental Discretization algorithm [39] that maps continuous gene expression values into discrete attributes. Interestingly, the algorithm applied to our dataset showed that the introduction of a single cutoff was sufficient to create two expression patterns, operationally defined as low and high, capable of describing the probe set status accurately enough for effective patients classification. This approach minimizes the variability and errors associated with the use of absolute values to interpret microarray gene expression data.

The validity of the discretization implemented by Rulex 2.0 was further documented by an empirical validation where we calculated the optimal cutoff value for each of the 11 probe sets tested in a Kaplan-Meier analysis of the patients' survival. It is noteworthy that such analysis utilized the survival time of the patient as opposed to 5 years survival considered by Rulex 2.0. Nevertheless, we demonstrated that the cutoff values calculated by either approaches were rather similar, thus supporting the robustness of the ADID algorithm to identify relevant discrete groups of expression values. From a technical point of view ADID is a multivariate method searching for the minimum number of cutoffs that separate patients belonging to different classes. On the other hand, the Kaplan-Meier scan is a univariate technique having the aim of identifying the value of the probe set that maximizes the distance among the survival times of resulting groups. It should be noted that these two approaches are independent from each other since they are based on different algorithms and different classifications.

Only 11 out of 62 probe sets of the original signature were considered by LLM for building the classifier. This selection has a biological meaning. In fact, the original 62 probe sets NB-hypo signature was obtained following a biology driven approach [1] in which the prior knowledge on tumor hypoxia was the bases for the analysis and the signature was derived from hypoxic neuroblastoma cell lines [14]. Hence, NB-hypo is optimized for detecting tumor hypoxia. The importance of hypoxia in conditioning tumor aggressiveness is documented by an extensive literature [17,19,20,22,59]. However, NB-hypo was not optimized to predict outcome that is dependent on factors other than hypoxia. Rulex 2.0 performed a feature selection by identifying the 11 probe sets that were the most relevant in predicting outcome among those of the NB-hypo signature.

One key feature of LLM is to implement an aggregative policy leading to the situation in which one patient can be covered by more than one rule. This leads to the advantage of avoiding dataset fragmentation typical of the divide-and-conquer paradigm. Furthermore, the robustness of the resulting model is increased; in fact, if a patient satisfies more than one rule for the same output class, the probability of a correct classification is higher.

The same outcome is generally predicted by every rule verified by a given patient. However, there are situations in which a patient satisfies rules associated with opposite outcomes, thus generating a potential conflict. Rulex 2.0 overcomes this problem by adopting a procedure for assigning a specific class on the basis of the characteristics of the verified rules. A conflict should not necessarily be considered as a limit of the proposed approach, but it could reflect a source of ambiguity present in the dataset. If this were the case, any method building models from data would always reflect this ambiguity.

The generation of a predictive classifier based on gene expression obtained from different institutions raised the question of a possible batch effect in the data. We utilized Frozen Surrogate Variable Analysis method, a batch effect removal method capable of estimating the training batch, and used it as a reference for adjusting batch effect of other batches. In particular, those for which no information about the outcome is known. This was not possible with other known batch removal methods such as the Combating Batch Effects (Combat) [60], which adjusts the expression values of both training and test batch [61]. We compared the performances achieved by ADID and LLM on the batch-adjusted dataset and those on the original dataset (no dataset modification) to measure the impact of batch effect on classifier performances. Performance obtained with the adjusted dataset showed that batch effect had negligible impact on performances. Previous studies observed that the application of batch effect removal methods for prediction does not necessarily result in a positive or negative impact [61]. Furthermore, batch effect removal methods may remove the true biologically based signal [61]. For this reasons, the analysis of the present manuscript was performed on the original dataset excluding any modification for batch removal.

The 9 rules generated by ADID and LLM achieved a good accuracy on an independent validation set. We compared the accuracy of our new classifier with that of the top performing classifiers for NB patients' outcome prediction listed in [13] to study the concordance of the performance achieved by the 9 rules with that of other previously published classifiers. The classifiers were generated on different signatures and algorithms and the accuracy reported ranged from 80% to 87%. Note that those classifiers were validated on a different test set; they utilized different algorithms and signatures. We concluded that the accuracy of our rules and that of other techniques reported in literature were concordant.

ADID represents an innovative discretization method that was used in combination with LLM for classification purposes. In the present study, ADID demonstrated to outperform other discretization algorithms, based on univariate analysis, indicating the capability of ADID to exploit the complex correlation structure commonly encountered in biomedical studies, including gene expression data sets. Moreover, ADID-LLM showed better performances with respect to the decision tree classifier. This was somewhat predictable because the lower performance of the decision tree with respect to other approaches has been pointed out in literature [15]. The LLM rules, SVM and PAM classifier achieved similar performances. The good performance achieved by ADID and LLM and the explicit representation of the knowledge extracted from the data provided by the rules demonstrated the utility of ADID and LLM in patients' outcome prediction.

Our dataset suffers of class-imbalance as many other datasets [48-53]. In fact, the good outcome class is over-expressed with respect to the poor outcome class. Rulex 2.0 implements a novel algorithmic strategy that allows setting up different weights to outcomes biasing the assignment to a class towards that of interest. It should be noted that weighting is effective only on class assignment for patients verified by conflicting rules. We have utilized the weight approach to represent the situation in which either poor or good outcome was favored or to address the imbalance between good and poor outcome patients in our dataset. We found that, in the absence of predefined weights, the algorithm generates a good performance somewhat unbalanced towards better classification of good outcome patients. By changing the weights we were in the position of steering the prediction towards a high precision in classifying poor outcome patients or in privileging the specificity. This tool may be or practical importance in the decision making process of clinicians that are confronted with difficult therapeutic choices.

## Conclusions

We provided the first demonstration of the applicability of data discretization and rule generation methods implemented in Rulex 2.0 to the analysis of microarray data and generation of a prognostic classifier. Rulex automatically derived a new signature, NB-hypo II, which is instrumental in predicting the outcome of NB patients. The performances achieved by Rulex are comparable and in some case better than those of other known data discretization and classification methods. Furthermore, the easy interpretability of the rules and the possibility to employ weighted classification make Rulex 2.0 a flexible and useful tool to support clinical decisions and therapy assignment.

## Methods

### Patients

Affymetrix GeneChip HG-U133plus2.0 enrolled 182 neuroblastoma patients on the bases of the availability of gene expression profile. Eighty-eight patients were collected by the Academic Medical Center (AMC; Amsterdam, Netherlands) [1,62]; 21 patients were collected by the University Children's Hospital, Essen, Germany and were treated according to the German Neuroblastoma trials, either NB97 or NB2004; 51 patients were collected at Hiroshima University Hospital or affiliated hospitals and were treated according to the Japanese neuroblastoma protocols [63]; 22 patients were collected at Gaslini Institute (Genoa, Italy) and were treated according to Italian AIEOP protocols. The data are stored in the R2 microarray analysis and visualization platform (AMC and Essen patients) or at the BIT-neuroblastoma Biobank of the Gaslini Institute. The investigators who deposited data in the R2 repository agree to use them for this work. In addition, we utilized data present on the public database at the Gene Expression Omnibus number GSE16237 for Hiroshima patients [63]. Informed consent was obtained in accordance with institutional policies in use in each country. In every dataset, median follow-up was longer than 5 years, tumor stage was defined as stages 1, 2, 3, 4, or 4s according to the International Neuroblastoma Staging System (INSS), normal and amplified MYCN status were considered and two age groups were considered, those with age at diagnosis smaller than 12 months and greater or equal to 12 months. Good and poor outcome were defined as the patient's status alive or dead 5 years after diagnosis. The characteristics of the patients are shown in Table 1.

### Batch effect measure and removal

The PVCA approach [41] was used to estimate the variability of experimental effects including batch. The pvca package implemented in R was utilized to perform the analysis setting up a pre- defined threshold of 60%. The analysis included Age at diagnosis, MYCN amplification, INSS stage, and Outcome and Institute variables. The estimation of experimental effects was performed before and after the batch effect removal.

The frozen surrogate variable analysis (FSVA) implemented in the sva package [64] was utilized for removing the batch effect from the training and the test sets. The parametric prior method and the Institute batch variable were set up for the analysis.

### Gene expression analysis

Gene expression profiles for the 182 tumors were obtained by microarray experiment using Affymetrix GeneChip HG-U133plus2.0 and the data were processed by MAS5.0 software according to Affymetrix guideline.

### Preprocessing step

To describe the procedure adopted to discretize values assumed by the probe sets a basic notation must be introduced. In a *classification *problem *d*-dimensional examples $x\in X\subset {ℜ}^{d}$, are to be assigned to one of *q *possible classes, labeled by the values of a categorical output *y*. Starting from a *training set S *including *n *pairs (***x****_i_, y_i_*), *i *= 1, ..., *n*, deriving from previous observations, techniques for solving classification problems have the aim of generating a model *g*(***x***), called *classifier*, that provides the correct answer *y = g*(***x***) for most input patterns ***x***. Concerning the components *x_j _*two different situations can be devised:

1. **ordered variables**: *x_j _*varies within an interval [*a,b*] of the real axis and an ordering relationship exists among its values.

2. **nominal (categorical) variables**: *x_j _*can assume only the values contained in a finite set and there is no ordering relationship among them.

A discretization algorithm has the aim of deriving for each ordered variable *x_j _*a (possibly empty) set of cutoffs γ*_jk_*, with *k *= 1, ..., *t_j_*, such that for every pair ***x****_u_*, ***x****_v _*of input vectors in the training set belonging to different classes (*y_u _≠ y_v_*) their discretized counterparts ***z****_u_*, ***z****_v _*have at least one different component.

Denote with ρ*_j _*the vector which contains all the α*_j _*distinct values for the input variable *x_j _*in the training set, arranged in ascending order, i.e. ρ*_jl_*<ρ*_j_*,_*l*+1 _for each *l *= 1, ..., α*_j_*-1. Then, we can consider a set of binary values τ*_jl_*, with *j *= 1, ..., *d *and *l *= 1, ..., α*_j_*-1, asserting if a separation must be set for the *j*-th variable between its *l*-th and (*l*+1)-th values:

$$\tau_{jl}=\left\{\begin{array}{cc} 1, & \text{if }\gamma_{\text{j}}\text{ contains }\rho_{\text{jl}}\\ 0, & \text{ otherwise} \end{array}\right)$$

Of course, the total number of possible cutoffs is given by

$$\overset{d}{\underset{j=1}{\sum }}\overset{\alpha_{j}-1}{\underset{l=1}{\sum }}\tau_{jl}$$

which must be minimized under the constraint that examples ***x****_u _*and ***x****_v _*belonging to different classes have to be separated at least by one cutoff. To this aim, let *X_juv _*the set of indexes *l *such that ρ*_jl _*lies between *x_uj _*and *x_vj_*:

$$X_{juv}=\left\{l|x_{uj}<\rho_{jl}<x_{vj}|\right\}$$

Then, the discretization problem can be stated as:

$$min_{\tau }\overset{d}{\underset{j=1}{\sum }}\overset{\alpha_{j}-1}{\underset{l=1}{\sum }}\tau_{jl}$$

(1) $$subj\;to\;\overset{d}{\underset{j=1}{\sum }}\;\underset{l\in X_{juv}}{\sum }\tau_{jl}\geq 1\;\text{for each }u,\;v,s.t.y_{u}\neq y_{v}$$

To improve the separation ability of the resulting set of cutoffs the constraint in (1) can be reinforced by imposing that

$$\overset{d}{\underset{j=1}{\sum }}\underset{l\in Xj_{uv}}{\sum }\tau_{jl}\geq s$$

for some *s *≥ 1. Intensive trials on real world datasets have shown that a good value for *s *is given by *s*= 0.2*d*; this choice has been adopted in all the analysis performed in the present paper.

Since the solution of the programming problem in (1) can require an excessive computational cost, a near-optimal greedy approach is adopted by the Attribute Driven Incremental Discretization (ADID) procedure [39]. It follows an iterative algorithm that adds iteratively the cutoff obtaining the highest value of a quality measure based on the capability of separating patterns belonging to different classes. Smart updating procedures enable ADID to efficiently attain a (sub) optimal discretization.

After the set of candidate cutoffs is produced, a subsequent phase is performed, to refine their position. This updating task significantly increases the robustness of final discretization.

### Classification by ADID and LLM implemented in Rulex 2.0

A classification model was built on the expression values of the 62 probe sets constituting NB-hypo signature [1]. Model generation and performance was established by splitting the dataset into a training set, comprising 60% of the whole patients cohort, and a test set comprising the remaining 40%. To build a classifier, a Rulex 2.0 process was designed. A discretizer component that adopts the Attribute Driven Incremental Discretization (ADID) procedure [39] and a classification component that adopts a rule generation method called Logic Learning Machine (LLM) were utilized into the process. Entropy based (EntMDL [42]), Modified Chi Square [43], ROC based (Highest Youden index ([44]), and Equal frequency (i.e. median expression for each feature) components have been executed as alternative discretization methods. To design the most accurate classifier one important parameter of the LLM component was evaluated. The parameter was the maximum error allowed on the training set. It defines the maximum percentage of examples covered by a rule with a class differing from the class of the rule. The parameter values evaluated ranged in the set 0%, 5%, 10%, 15%, 20%, 25%, and 30%. For each parameter value, a 10 times repeated 10-fold cross validation analysis was performed and the classification performances were collected. The parameters' choice that obtained the best mean classification accuracy was selected to train the final gene expression based classifier on the whole training set utilizing the aforementioned Rulex components. The *Rulex *software suite is commercialized by Impara srl[40].

Decision Tree [45], Support Vector Machines (SVM) [46], and Prediction Analysis of Microarrays (PAM) [47]were run on the same training and test sets for reference.

### Performance evaluation in predicting patients' outcome

To evaluate the prediction performance of the classifiers we used the following metrics: accuracy, recall, specificity and negative predictive values (NPV), considering good outcome patients as positive instances and poor outcome patients as negative instances. Accuracy is the proportion of correctly predicted examples in the overall number of instances. Recall is the proportion of correctly predicted positive examples against all the positive instances of the dataset. Precision is the proportion of correctly classified positive examples against all the predicted positive instances. Specificity is the proportion of correctly predicted negative examples against all the negative instances of the dataset. NPV is the proportion of the correctly classified negative examples against all the predicted negative instances.

### Rule quality measures

*Rule generation methods *constitute a subset of classification techniques that generate explicit models *g*(***x***) described by a set of *m *rules *r_k_, k *= 1, ..., *m*, in the **if-then **form:

if < premise > then < consequence > 

where <*premise*> is the logical product (**and**) of *m_k _*conditions *c_kl_*, with *l *= 1, ..., *m_k_*, on the components *x_j_*, whereas <*consequence*> gives a class assignment y=ỹ for the output. In general, a condition *c_kl _*in the premise involving an ordered variable *x_j _*has one of the following forms *x_j _*>λ, *x_j _≤ μ*, λ<*x_j _≤ μ*, being λ and *μ *two real values, whereas a nominal variable *x_k _*leads to membership conditions $x_{k}\in \left\{\alpha ,\;\delta ,\;\sigma \right\}$, being *α, δ*, σ admissible values for the *k*-th component of ***x***.

For instance, if *x*_1_ is an ordered variable in the domain {1, ..., 100} and *x*_2 _is a nominal component assuming values in the set {*red, green, blue*}, a possible rule *r*_1 _is

$$if\;x_{\text{1}}>\text{4}0\;and\;x_{\text{2}}\in \left\{red,blue\right\}then\;y=0$$

where 0 denotes one of the *q *possible assignments (classes).

According to the output value included in their consequence part, the *m *rules *r_k _*describing a given model *g*(***x***) can be subdivided into *q *groups *G*_1_, *G*_2_, ..., *G_q_*. Considering the training set *S*, any rule *r*∈*G_l _*is characterized by four quantities: the numbers *TP*(*r*) and *FP*(*r*) of examples (***x****_i_, y_i_*) with *y_i _= y_l _*and *y_i _*≠ *y_l_*, respectively, that satisfy all the conditions in the premise of *r*, and the numbers *FN*(*r*) and *TN*(*r*) of examples (***x****_i_, y_i_*) with *y_i _= y_l _*and *y_i _*≠ *y_l_*, respectively, that do not satisfy at least one of the conditions in the premise of *r*.

The quality of a rule was measured utilizing the following quantities. Give a rule *r*, we define the *covering C*(*r*), the *error E*(*r*), and the *precision P*(*r*) according to the following formulas:

$$C\left(r\right)=\frac{TP\left(r\right)}{TP\left(r\right)+FN\left(f\right)}\;\;,\;\;E\left(r\right)=\frac{FP\left(r\right)}{FP\left(r\right)+TN\left(r\right)},\;\;P\left(r\right)=\frac{TP\left(r\right)}{TP\left(r\right)+FP\left(r\right)}$$

The covering of a rule is the fraction of examples in the training set that satisfy the rule and belong to the target class. The error of a rule is the fraction of examples in the training set that satisfy the rule and do not belong to the target class. The precision of a rule is the fraction of examples in the training set that do not belong to the target class but satisfy the premises of the rule. The greater was the covering and the precision, the higher was the generality and the correctness of the corresponding rule.

To test the statistical significance of the rules we used a Fisher's exact test (FET) implemented by the software package R. The test of significance considered significant any rule having P < 0.05.

### Relevance measure and ranking of the probe sets

To obtain a measure of importance of the features included into the rules and rank these features according to this value, we utilized a measure called Relevance *R*(*c*) of a condition *c*. Consider the rule *r' *obtained by removing that condition from *r*. Since the premise part of *r' *is less stringent, we obtain that *E*(*r'*) ≥ *E*(*r*) so that the quantity*R*(*c*) = (*E*(*r'*)−*E*(*r*))*C*(*r*) can be used as a measure of relevance for the condition *c *of interest.

Since each condition *c *refers to a specific component of ***x***, we define the relevance *R_v_*(*x_j_*) for every input variable *x_j _*as follows:

$$R_{v}\left(x_{j}\right)=1-\underset{k}{\prod }\left(1-R\left(c_{kl}\right)\right)$$

where the product is computed on the rules *r_k _*that includes a condition *c_kl _*on the variable *x_j_*.

Denote with *V_kl _*the attribute involved in the condition *c_kl _*of the rule *r_k _*and with *S_kl _*the subset of values of *V_kl _*for which the condition *c_kl _*is verified. If *V_kl _*is an ordered attribute and the condition *c_kl _*is *V_kl _≤ a *for some value *a*∈*S_kl_*, then the contribution to *R_v_*(*x_j_*) is negative. Hence, by adding the superscript − (resp. +) to denote the attribute *V_kl _*with negative (resp. positive) contribution, we can write *R_v_*(*x_j_*) for an ordered input variable *x_j _*in the following way:

$$R_{v}\left(x_{j}\right)=\underset{V_{kl}}{\prod }\left(1-R\left(c_{kl}\right)\right)-\underset{V_{kl}}{\prod }\left(1-R\left(c_{kl}\right)\right)$$

where the first (resp. second) product is computed on the rules *r_k _*that includes a condition *c_kl_*

leading to a negative (resp. positive) contribution for the variable *x_j_*.

### Output assignment for a new instance

When the model g(**x**) described by the set of m rules r_k_, k = 1, ..., m, is employed to classify a new instance **x**, the <premise> part of each rule is examined to verify if the components of **x **satisfy the conditions included in it. Denote with Q the subset of rules whose <premise> part is satisfied by **x**; then, the following three different situations can occur:

1. The set Q includes only rules having the same output value *ỹ *in their <consequence> part; in this case the class *ỹ *is assigned to the instance **x**.

2. The set Q contains rules having different output values in their <consequence> part; it follows that Q can be partitioned into q disjoint subsets Q_i_, (some of which can be empty) including the rules r pertaining to the ith class. In this case, to every attribute x_j _can be assigned a measure of consistency t_ij _given by the maximum of the relevance r(c) for the conditions c involving the attribute x_j _and included in the <premise> part of the rules in Q_i_. Then, to the instance **x **is assigned the class  ỹ associated with the following maximum:

$$ỹ=\underset{i=1,\cdots q}{\text{argmax}}\overset{d}{\underset{i=1}{\sum }}t_{ij}$$

3. The set *Q *is empty, i.e. no rule is satisfied by the instance ***x***; in this case the set *Q*_−1_ containing the subset of rules whose <*premise*> part is satisfied by ***x ***except for one condition is considered and points 1 and 2 are again tested with *Q = Q_−_*_1_. If again *Q *is empty the set *Q*_−2 _containing the subset of rules whose <*premise*> part is satisfied by ***x ***except for two conditions is considered and so on.

The conflicting case 2 can be controlled in Rulex 2.0 by assigning a set of weights *w_i _*to the output classes; in this way equation (1) can be written as

$$ỹ=\underset{i=1,\cdots q}{\text{argmax}}\overset{d}{\underset{i=1}{\sum }}t_{ij}w_{i}$$

and we can speak of weighted classification.

## List of abbreviations

INSS: International Neuroblastoma Staging System; FET: Fisher's Exact test; NPV: Negative Predictive Value; INRG: International Neuroblastoma Risk Group; LLM: Logic Learning Machine; SNN: Switching Neural Networks; SC: Shadow Clustering; TP: true positives; FP: false positives; TN: true negatives; FN: false negatives; NB: neuroblastoma; ADID: Attribute Driven Incremental Discretization; WCS: weighted classification system; PVCA: principal variance component analysis; SVA: surrogate variable analysis; FSVA: frozen surrogate variable analysis.

## Competing interests

The authors declare that they have no competing interests.

## Authors' contributions

DC conceived the project, performed the statistical analysis and drafted the manuscript. MM suggested the use of LLM, designed some of the experiments, designed the Rulex software and helped to draft the manuscript. SP performed computer experiments and helped to draft the manuscript, RV and MC, participated to the development of the project. FB and PB carried out the microarray data analysis. LV supervised the study and wrote the manuscript.

## Supplementary Material

Additional file 1 **Title of data: Batch effect and LLM prediction performance. Description of data: the file contains a table showing the influence of batch effect on LLM prediction performance. Additional file 1. Table 1. *Influence of batch effect on LLM prediction performance*. The table shows the influence of batch effect calculated on accuracy, recall, precision, and specificity and NPV measures. Performances are comparable removing batch effect from the dataset**. Click here for file

## Declarations

Charge for this article was paid by a grant of the Fondazione Italiana per la Lotta al Neuroblastoma.

This article has been published as part of *BMC Bioinformatics *Volume 15 Supplement 5, 2014: Italian Society of Bioinformatics (BITS): Annual Meeting 2013. The full contents of the supplement are available online at http://www.biomedcentral.com/bmcbioinformatics/supplements/15/S5
