# Supplementary material for: Use of Attribute Driven Incremental Discretization and Logic Learning Machine to build a prognostic classifier for neuroblastoma patients
Source: BMC Bioinformatics. 2014 May 6;15(Suppl 5):S4. doi: 10.1186/1471-2105-15-S5-S4 (PMC4095004; doi:10.1186/1471-2105-15-S5-S4)
Supplement: Additional file 1 — Title of data: Batch effect and LLM prediction performance. Description of data: the file contains a table showing the influence of batch effect on LLM prediction performance. Additional file 1. Table 1. Influence of batch effect on LLM prediction performance. The table shows the influence of batch effect calculated on accuracy, recall, precision, and specificity and NPV measures. Performances are comparable removing batch effect from the dataset. [file 1471-2105-15-S5-S4-S1.pdf]

Additional file 1

Table 1 - Influence of batch effect on LLM prediction performance

Dataset modification[f]	Accuracy[a]	Recall[b]	Precision[c]	Specificity[d]	NPV[e]
**None**	80%	90%	82%	57%	72%
**FSVA Batch adjusted[f]**	74%	78%	83%	65%	58%

[a] Accuracy is the fraction of correctly classified patients and overall classified patients.

[b] Recall is the fraction of correctly classified good outcome patients and the overall predicted good outcome patients

[c] Precision is the fraction of correctly classified good outcome patients and the predicted good outcome patients.

[d] Specificity is the fraction of correctly classified poor outcome patients and the overall poor outcome patients

[e] NPV (Negative predictive value) is the fraction of correctly classified poor outcome patients and the overall predicted poor outcome patients

[f] The dataset was modified according to the FSVA batch effect removal method described in the Materials and Methods.
